# DNA barcoding for identification of fish species in the Taiwan Strait

**DOI:** 10.1371/journal.pone.0198109

**Published:** 2018-06-01

**Authors:** Xing Bingpeng, Lin Heshan, Zhang Zhilan, Wang Chunguang, Wang Yanguo, Wang Jianjun

**Affiliations:** Laboratory of Marine Biology and Ecology, Third Institute of Oceanography State Oceanic Administration, Xiamen, Fujian, China; Chang Gung University, TAIWAN

## Abstract

DNA barcoding based on a fragment of the cytochrome c oxidase subunit I (COI) gene in the mitochondrial genome is widely applied in species identification and biodiversity studies. The aim of this study was to establish a comprehensive barcoding reference database of fishes in the Taiwan Strait and evaluate the applicability of using the COI gene for the identification of fish at the species level. A total of 284 mitochondrial COI barcode sequences were obtained from 85 genera, 38 families and 12 orders of fishes. The mean length of the sequences was 655 base pairs. The average Kimura two parameter (K2P) distances within species, genera, families, orders and classes were 0.21%, 6.50%, 23.70% and 25.60%, respectively. The mean interspecific distance was 31-fold higher than the mean intraspecific distance. The K2P neighbor-joining trees based on the sequence generally clustered species in accordance with their taxonomic classifications. High efficiency of species identification was demonstrated in the present study by DNA barcoding, and we conclude that COI sequencing can be used to identify fish species.

## Introduction

More than 30,000 species of fish exist worldwide, accounting for more than half of all vertebrates. Aside from being an important component of biodiversity, fish also possess direct economic value and are important animal protein sources for humans[1, 2]. The classification and identification of fish is not only the subject of taxonomy studies but also the key to fishery investigations, the assessment of nature reserves and the identification of food and drug ingredients[3, 4]. The identification of fish species also mainly relies on morphometric and meristic characters[5]. Fish have remarkable diversity of morphological characteristics, and most fish go through ontogenetic metamorphism. Many morphometric characteristics change during the stages of ontogenetic development[6]. Convergent and divergent adaptation also lead to changes in the morphological characteristics of fish species, imposing great challenges to morphological taxonomy, in which species identification is mainly based on morphological characteristics, and the classification of many species has thus also been controversial[7]. The limitations inherent in morphology-based identification systems and the declining number of taxonomists call for a molecular approach to identify species[6, 8].

DNA sequence analysis has been used to assist species identification with the development of molecular biology. However, the accuracy of molecular identification relies on having a reliable and complete reference database, as inconsistent genetic marker usage could impede the application of molecular authentication[9]. Different DNA markers have been used in different taxonomic groups. In 2003, Hebert et al.[10] proposed DNA barcoding technology, in which the mitochondrial cytochrome c oxidase subunit I (COI) gene sequence was used as a barcode for species identification with the expectation of barcoding all species for the purpose of species identification and classification. It was found that the intraspecific diversity of the COI gene in animals was significantly lower than the interspecific diversity, using the COI gene as a barcode was effective for classifying and identifying vertebrates and invertebrates, and the COI gene has been widely used in various biological groups[11–14]. Compared with the traditional morphological classification methods, the advantages of the DNA barcoding technology are mainly as follows: 1) Some species have extremely similar external morphological characteristics; therefore, it is difficult to distinguish them from each other merely by morphological characteristics. DNA barcoding technology can help accurately distinguish such species. 2) Morphological differences can vary considerably during various developmental stages, but individuals at different developmental stages can be identified by the DNA barcoding technology. 3) The DNA barcoding technology can allow for the discovery of cryptic species. Cryptic species are two or more species that are morphologically similar yet genetically distinct. Because of their morphological similarities, cryptic species are identified as the same species in the existing system. Using DNA barcoding technology, the large molecular evolutionary distance between these species can be revealed, thereby discovering cryptic species.

The Taiwan Strait belongs to the shallow sea of the subtropical continental shelf and is the passage between the East China Sea and the South China Sea. The sea area of the Taiwan Strait has a complex physical and chemical environment and is influenced by the Kuroshio tributaries, the drifting of the South China Sea monsoon and the coastal flows in Fujian and Zhejiang Provinces, China. The Taiwan Strait has rich fishery resources and is one of the most important fishing grounds off the coast of China. The Taiwan Strait has been drawing much attention due to its unique geographical location and marine environment characteristics, and studies on its ecosystem have gained attention. As human activities have intensified, over-fishing, habitat destruction and climate change have generated significant impacts on the biodiversity and structure of the fish community in the Taiwan Strait. The decline in genetic variation of a population diminishes the ability of fish to adapt to environmental changes and decreases their chances of long-term survival[3]. To promote sustainability, better control and management of fisheries should be implemented. The identification of fish species still stands as one of the most basic but important issues in fisheries management. In the present study, we examined COI diversity within and among 85 fish species, most of which were commercial species, with the goal of testing the utility of DNA barcoding as a tool to identify fish species. The DNA barcode records generated in this study will be available to researchers to monitor and conserve the fish diversity in this region.

## Materials and methods

### Ethics statement

All fish species were caught in the offshore area (not national parks, other protected areas, or private areas, etc.), so no specific permissions were required for these locations/activities. Ethical approval was not required for this study because no endangered or protected fish species were involved. Specimen collection and maintenance were performed in strict accordance with the recommendations of Animal Care Quality Assurance in China.

### Sample collection

**Fig 1:**
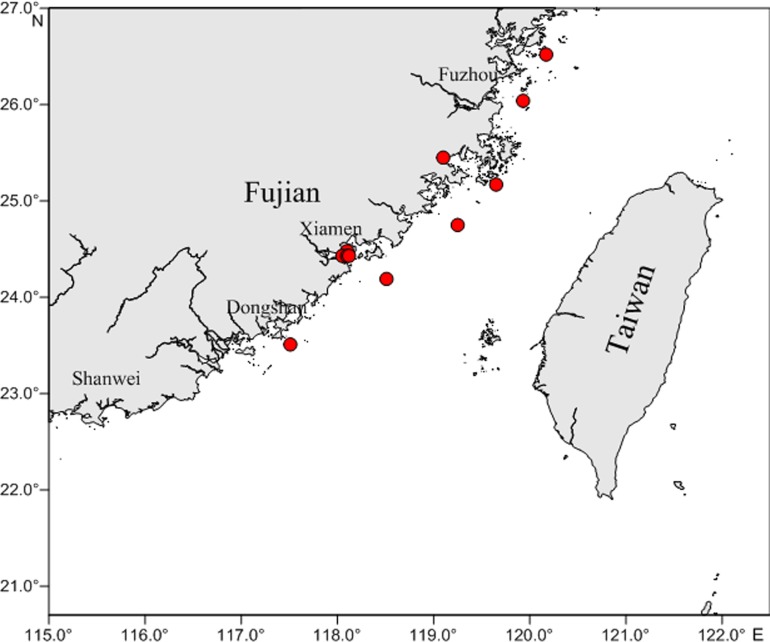
Distribution of the sampling localities for the specimens collected in this study.

All fish specimens were captured with a drawl net at nine locations in the Taiwan Strait (Fig 1, Table 1). All specimens were morphologically identified by experts and taxonomists, who mainly followed the identification keys of Liu et al. (2013)[15]. A total of 284 fish samples was chosen for the research, and the mean number of individuals per species was 3. The voucher specimens were fixed with 95% ethanol and deposited in the Marine Biological sample Museum at the Third Institute of Oceanography, State Oceanic Administration. After morphological examination, muscle tissue samples were dissected from each specimen and stored in 95% ethanol at −20°C.

**Table 1:** Classification of species analyzed for COI sequences and sequence source.

Order	Family	Genus/Species	GenBank accession numbers					
Anguilliformes	Ophichthidae	*Scolecenchelys macroptera*	KX215169	KY472811	KY472810			
		*Muraenichthys gymnopterus*	KX215178	KX215179	KX215180			
		*Ophichthus apicalis*	KX215176	KX215177	KY472814			
		*Ophichthus brevicaudatus*	KX215170	KX215171	KY472812			
		*Ophichthus celebicus*	KX215188	KX215189	KY472819			
		*Pisodonophis cancrivorus*	KX215181	KX215182	KY472817			
		*Pisodonophis boro*	KX215190	KX215191				
		*Apterichtus klazingai*	KX215197	KX215198				
		*Neenchelys parvipectoralis*	KX215185	KX215186	KX215187			
		*Cirrhimuraena chinensis*	KX215192	KX215193	KX215194			
	Congridae	*Uroconger lepturus*	KX215172	KX215173	KX215174	MG220575		
	Muraenidae	*Gymnothorax reticularis*	KX215183	KX215184	MG220570			
	Muraenesocidae	*Muraenesox cinereus*	KX215195	KX215196				
Siluriformes	Ariidae	*Netuma thalassina*	KP260470	KX254510	KX254511	KX254512	MG220574	EF607325*
	Plotosidae	*Plotosus lineatus*	MG220589	EU595237*				
Aulopiformes	Synodontidae	*Saurida elongate*	KP260467	MG574518	MG574519	EF607514*		
		*Saurida macrolepis*	KP260474	MG574444	MG574445			
		*Harpadon nehereus*	KP260457	KX254522	KX254523	KX254524	MG574499	JN242630*
Lophiiformes	Antennariidae	*Antennarius striatus*	MG220554	MG220555	MG220556	AB282828*		
Perciformes	Polynemidae	*Polynemus sextarius*	KX254537	KX254538	KX254539	MG574490	MG574491	FJ238014*
Clupeiformes	Engraulidae	*Coilia mystus*	MG220577	KF056322*	KY223613*			
		*Thryssa dussumieri*	MG220572	MG574430	MG574431	AP017953*		
		*Thryssa kammalensis*	KP260469	KP260453	KX254519	KX254520	KX254521	KX227717*
			KT985048*					
		*Thryssa hamiltonii*	JQ681498*	KX096870*				
		*Coilia grayii*	KX254516	KX254517	KX254518	MG574523	KP317088*	
		*Stolephorus commersonnii*	KX254525	KX254526	KX254527	MG574496	KM236095*	
		*Engraulis japonicas*	MG220580	MG637134	MG637135	AP017957*		
		*Setipinna tenuifilis*	MG220585	MG637137	MG637138	KP403942*		
	Pristigasteridae	*Ilisha elongate*	KP260471	KX254506	KX254507	KX254508	KX254509	MG574531
			AP009141*					
		*Sardinella zunasi*	KX254478	KX254479	MG574480	MG574481	JF952838*	
		*Sardinella lemuru*	KX254484	KX254485	KX254486	MG574447	HQ231366*	
Gasterosteiformes	Pegasidae	*Pegasus laternarius*	MG220582	MG574469	MG574470			
Syngnathiformes	Syngnathidae	*Syngnathus schlegeli*	KP260464	MG637143	MG637144	KP861226*		
	Fistulariidae	*Fistularia commersonii*	KP260463	MG637140	MG637141	AP005987*		
Scorpaeniformes	Platycephalidae	*Inegocia cf*. *japonica*	MG220550	MG220551	MG637146	JX488180*		
		*Kumococius rodericensis*	MG220564	MG574426	MG574427	EF607413*		
	Scorpaenidae	*Scorpaena miostoma*	MG220568	MG637149	MG637150	KU199087*		
Perciformes	Carangidae	*Alepes kleinii*	KX254480	KX254481	MG637152	MG637153	KF728081*	
		*Decapterus maruadsi*	KP260472	MG637155	MG637156	MG637157	KU302323*	
		*Parastromateus niger*	MG220581	MG637159	MG637160	MG637161	KJ192332*	
		*Megalaspis cordyla*	MG220590	MG637162	MG637163	MG637164	KM522836*	
	Eleotridae	*Butis koilomatodon*	KX254490	KX254491	MG574472	MG574473		
	Gobiidae	*Myersina filifer*	KX254492	KX254493	MG574549	MG574550	KU216079*	
		*Chaeturichthys stigmatias*	KX254494	KX254495	MG574459	G574460	G574461	KU199164*
		*Parachaeturichthys polynema*	MG220548	MG220549	MG637166	KY315375*		
		*Tridentiger barbatus*	KX254499	KX254500	MG574545			
		*Tridentiger trigonocephalus*	KX254503	KX254504	KX254505	KT282115*		
		*Trypauchen vagina*	KP260458	KX254496	KX254497	KJ865406*		
	Amblyopinae	*Taenioides anguillaris*	KX254501	KX254502	MG574538	KT188772*		
		*Odontamblyopus rubicundus*	KP260455	MG637167	MG637168	MG637169	KY315378*	
	Siganidae	*Siganus fuscescens*	KX254513	KX254514	KX254515	MG574525	KT943386*	
	Sillaginidae	*Sillago sihama*	KX254528	KX254529	KX254530	MG574493	KP112426*	
		*Sillago japonica*	KX254531	KX254532	KX254533	MG637171	HM180882*	
	Sciaenidae	*Collichthys lucidus*	KX254534	KX254535	KX254536	MG220565	MG574442	JN857362*
		*Pennahia anea*	MG574451	MG574452	MG574453	KY371940*		
		*Pennahia argentata*	MG574462	MG574463	MG574464	HQ890946*		
		*Dendrophysa russelii*	MG574455	MG574456	MG574457	JQ728562*		
		*Larimichthys crocea*	MG574434	MG574435	MG574436	KP112392*		
		*Larimichthys polyactis*	MG574438	MG574439	MG574440	HM068242*		
	Lateolabracidae	*Lateolabrax japonicus*	KX254540	KX254541	KX254542	MG574487	KX254540*	
		*Epinephelus quoyanus*	KP260477	MG637173	MG637174	KC790539*		
		*Epinephelus awoara*	KP260461	MG637177	MG637178	JX109835*		
	Leiognathidae	*Leiognathus brevirostris*	KX254543	KX254544	KX254545			
		*Nuchequula nuchalis*	KP260478	MG574483	MG574484	AB355911*		
		*Secutor ruconius*	KP260454	MG574506	MG574507	MG574508	EF607542*	
		*Photopectoralis bindus*	MG220584	MG637179	MG637180	MG637181	EF607421*	
	Callionymidae	*Callionymus formosanus*	MG220573	MG637183	MG637184	MG637185	KP267580*	
		*Callionymus curvicornis*	MG220552	MG220553		KY371260*		
	Centrolophidae	*Psenopsis anomala*	KP260466	MG637187	MG637188	HM180801*		
	Terapontidae	*Terapon jarbua*	KP260475	KX254487	KX254488	KP455734*		
	Scatophagidae	*Scatophagus argus*	KP260476	KX254482	KX254483	KC790398*		
	Gerreidae	*Gerres limbatus*	KX254472	KX254473	KX254474	EF607391*		
	Scombridae	*Scomberomorus commerson*	MG220579	MG637191	MG637192	KP267578*		
	Trichiurinae	*Lepturacanthus savala*	KP260456	MG220578	MG637195	JN990858*		
	Hapalogenyidae	*Hapalogenys analis*	KP260460	MG220569	MG637197	HM180600*		
		*Hapalogenys nigripinnis*	MG574551	MG574552	MG574553	JN242701*		
Pleuronectiformes	Paralichthyidae	*Paralichthy solivaceus*	KX254475	KX254476	KX254477	AB028664*		
	Soleidae	*Zebrias zebrinus*	MG220587	MG637199	MG637200	MG637201	KC491209*	
		*Cynoglossus abbreviatus*	MG574476	MG574477	MG574478	GQ380410*		
		*Pseudaesopia japonica*	MG574502	MG574503	MG574504	KJ433568*		
	Pleuronectinae	*Pleuronichthys cornutus*	KP260473	MG637203	MG637204	JQ639071*		
Tetraodontiformes	Tetraodontidae	*Takifugu fasciatus*	KP260468	MG574465	MG574466	GQ409967*		
		*Takifugu oblongus*	KP260459	MG574510	MG574511	MG574512	KT364766*	
		*Takifugu xanthopterus*	MG574556	MG574557	MG574558	MG574559	JQ681824*	
		*Takifugu poecilonotus*	MG574514	MG574515	MG574516	JQ681821*		
	Monacanthidae	*Paramonacanthus sulcatus*	MG220583	MG637207	MG637208	EF607467*		

‘*’ the sequence was downloaded from NCBI

Total DNA was extracted from a small piece of ethanol-preserved tissue according to the standard DNA barcoding methods for fish[2]. Approximately 655 bp were amplified from the 5’ region of the COI gene employing the primers described in Ward et al.[2]:

FishF1-5′TCAACCAACCACAAAGACATTGGCAC3′;

FishF2-5′TCGACTAATCATAAAGATATCGGCAC3′;

FishR1-5′TAGACTTCTGGGTGGCCAAAGAATCA3′;

FishR2-5′ACTTCAGGGTGACCGAAGAATCAGAA3′.

The amplification reaction was performed in a total volume of 25 μl, including 16.25 μl ultrapure water, 2.25 μl 10× PCR buffer, 1.25 mM MgCl_2_, each dNTP at 0.2 mM, each primer at 2 mM, 1.25 U Taq DNA polymerase and 1 μl DNA template. The thermal cycling conditions consisted of an initial step of 2 min at 95°C followed by 35 cycles of denaturing (94°C, 30 s), annealing (54°C, 30 s) and extension (72°C, 1 min), with a final extension at 72°C for 10 min; the samples were then held at 4°C. The PCR samples were screened for the existence of PCR products on a 1.0% agarose gel. Sequencing in both directions was performed by Sangon Biotech (Shanghai).

### Data analyses

Sequences were manually edited using the SeqMan program (DNAStar software) combined with manual proofreading; each base of the spliced sequences was ensured to be correct before submitting them to GenBank (Table 1). Next, the sequences were aligned using ClustalW in MEGA 6.0 software, and parameters including the sequence length, GC content, polymorphic loci and parsimony informative sites were calculated. The distances within species and between species were calculated using the Kimura-2-parameter (K2P) model[16]; a phylogenetic tree was constructed using the neighbor-joining (NJ) method. The clade credibility in the tree that was obtained using the NJ method was tested by bootstrapping, in which 1000 repeated sampling tests were performed to obtain the support values of the clade nodes.

## Results

A total of 284 mitochondrial COI barcode sequences were obtained from 85 genera, 39 families and 11 orders of fishes (GenBank accession numbers and taxonomic data are listed in Table 1). After editing, the consensus length of all barcode sequences was 655 bp, and no stop codons, insertions or deletions were observed in any of the sequences. All analyzed sequences were larger than 600 bp.

Nucleotide pair frequency analysis of the entire dataset revealed that 325 of 655 (49.62%) sites were conserved, 330 of 655 (50.38%) sites were variable, 330 of 655 (56.27%) sites were parsimony informative, and no singleton sites were present. The average number of identical pairs (ii) was 519, of which 202, 216 and 101 were found at the first, second and third codon positions, respectively. Transitional pairs (si = 76) were found to be more common than transversional pairs (sv = 60), with a si/sv (R) ratio of 1.27 for the dataset. Both transitional and transversional pairs were most common at the third codon position (si = 62 and sv = 56).

The overall mean nucleotide base frequencies observed for these sequences were T (28.90%), C (28.40%), A (24.30%) and G (18.40%). The base composition analysis for the COI sequence showed that the average T content was the highest and the average G content was the lowest; the AT content (53.20%) was higher than the GC content (46.80%). The GC contents at the first, second and third codon positions for the 11 sole fish were 56.70%, 43.10% and 40.60%, respectively (Table 2). Of these, the GC content at the first codon position was the highest, which can be attributed to base usage bias among the three codon positions. The usage frequency of only C was similar among the three codon positions, whereas the other three bases had significantly different usage frequencies. At the first codon position, the usage of T (18.00%) was the lowest, and the usages of the other bases were C (25.60%), A (25.60%) and G (31.10%). At the second codon position, the content of T (42.00%) was highest, and the contents of the other bases were C (28.40%), A (15.00%) and G (14.70). At the third codon position, the base usage was T (27.00%), C (31.10%), A (32.20%) and G (9.50%); the G content was the lowest, exhibiting a clear pattern of anti-G bias.

**Table 2:** GC content of each order and the first, second and third codon positions.

GC% Order	Total	1st	2nd	3rd
**Pleuronectiformes**	46.8	55.8	42.6	42
**Tetraodontiformes**	47.9	57.8	43.1	42.6
**Scorpaeniformes**	45.8	55.9	43.1	38.2
**Gasterosteiformes**	47.5	55.7	43	43.7
**Clupeiformes**	46.4	57.1	43.1	38.9
**Mugiliformes**	50.4	58.2	43.1	49.7
**Lophiiformes**	41.9	54.6	43.1	28.3
**Myctophiformes**	50.1	56.9	43.1	50.5
**Siluriformes**	47.8	57.8	43	42.6
**Anguilliformes**	46.5	57.1	43.1	39.2
**Perciformes**	46.6	56.7	43.2	40
**Avg.**	46.8	56.7	43.1	40.6

The Kimura-2-parameter model is recommended by the Consortium for the Barcode of Life (CBOL) for calculating genetic distance [16, 17]. In this study, the Kimura-2-parameter model was used to calculate the genetic distances within and between species for the fish used in this study. As shown in Table 3, the K2P distances of the COI sequence within species ranged from 0 to 1.83%, with an average distance of 0.21%; the largest distance of 1.83% was found in *Terapon jarbua*, and the distances for all remaining species were less than 1%. The genetic distances between species ranged from 0 to 21.70%, with an average of 6.50%, which was 31 times the average genetic distance within species. The genetic distance between genera was 7.70–30.50%, with an average of 23.70%, and the genetic distance between families was 17.60–31.80%, with an average of 25.60%. Only genetic distances within species were less than 2%, and the mean genetic distances between species, between genera and between families were all greater than 5%, which were much higher than the distances within species. The data also show that the genetic distance (K2P) was larger at higher taxonomic levels, and the increases in genetic distances (K2P) above the species level were smaller and less pronounced at higher taxonomic levels.

**Table 3:** Genetic divergence (percentage, K2P distance) within various taxonomic levels.

Comparisons within	taxa	Mean (%)	Minimum (%)	Maximum (%)	S.E.*
Species	85	0.21	0	1.83	0.01
Genus	68	6.50	0	21.70	0.02
Family	39	23.70	7.70	30.50	0.02
Order	11	25.60	17.60	31.80	0.02

* Standard error.

**Fig 2:**
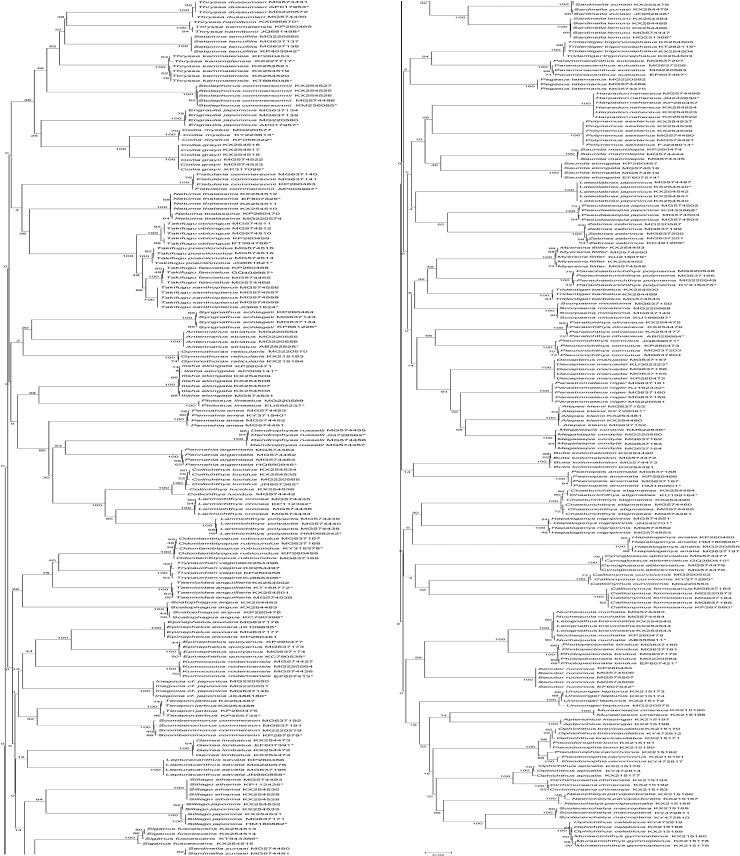
Neighbor-joining tree based on COI sequences using K2P distances.

The NJ tree, including 284 species with all haplotypes and 71 species from NCBI (Table 1), is provided in Fig 2. Most of the specimens of the same species were clustered together, which reflected the prior taxonomic assignment based on morphology. No taxonomic deviation was detected at the species level, indicating that the majority of the examined species could be authenticated by the barcode approach.

## Discussion

DNA barcoding technology allows for species identification by taking advantage of a DNA sequence fragment that is shared by organisms that have significant interspecies differences. This technology breaks through the over-reliance on the personal abilities and experiences of taxonomists in traditional morphological classification and enables the informatization and standardization of species identification. The mitochondrial COI gene, which exhibits high levels of conservation within species and modest levels of genetic variability between different species, is usually utilized as a species barcode, and its high efficiency in species identification has been reported in Japanese marine fishes [6], Indian freshwater fishes[18], Taiwan ray-finned fishes[9] and Mediterranean fishes[19]. In this study, we successfully amplified the COI barcode sequences for 89 marine fish species. The primer pairs used in this study could amplify the target region without any deletions or insertions, indicating that DNA barcoding could be used as a global standard for identifying fish species. All barcode sequences were a consensus length of 655 bp, no stop codons were detected, and the sequences were free of nuclear mitochondrial pseudogenes (NUMTs). Vertebrate NUMTs are typically smaller than 600 bp [2, 20]. The presence of NUMTs can cause misestimation of the biodiversity. The number of COI genes is greater than that of pseudogenes, so conserved primers should preferentially amplify mitochondrial DNA over NUMTs, and there was no evidence of NUMTs in the fish. A nucleotide pair frequency analysis resulted in 325 conserved sites, 330 variable sites, 330 bp parsimony informative sites and no singleton sites. There were more transitional pairs (si = 76) than transversional pairs (sv = 60). The observed nucleotide pair frequencies were similar to those reported in studies of fishes in Turkey. Both transitional and transversional pairs were highest at the third codon position (62 and 56 for si and sv, respectively), and synonymous mutations mostly occurred at the third position. The amount of variation observed in mitochondrial DNA can lead to demographic changes in fish populations.

The base composition analysis of the COI sequence revealed AT content (53.20%) that was higher than GC content (46.80%), which is a result similar to the results found in Australian[2], Canadian [21] and Cuban fish species[22]. The GC contents in the first, second and third codon positions for the 11 sole fish were 56.70%, 43.10% and 40.60%, respectively. Of these, the GC content of the first codon position was significantly higher than those of the other two positions, which can be attributed to base usage bias at the three codon positions. At the first codon position, the usage of T (18.00%) was the lowest, and the usage of the other bases was C (25.60%), A (25.60%) and G (31.10%). At the second codon position, the usage of T (42.00%) was the highest, and the usage of the other bases was C (28.40%), A (15.00%) and G (14.70%). At the third codon position, the base usage was T (27.00%), C (31.10%) and A (32.20%), each of which was higher than G (9.50%). The third codon position had a clear pattern of anti-G bias, and similar patterns have also been observed in Ophichthyidae and Soleidae [23, 24]. In species evolution, the codon positions of mitochondrial genes are subjected to varying degrees of base-mutation selection pressure, and base usage bias may be caused by base-mutation pressure in codon positions.

The efficiency of species identification through DNA barcoding depends on both interspecific divergence and intraspecific divergence. Barcode analysis attempts to identify the boundaries to delineate species, which corresponds to the divergence between the nearest neighbors within a group[10, 18]. However, there is still no universal standard threshold defined for interspecies demarcation. The difference between minimum congeneric and maximum conspecific divergence was recently used to define the barcoding gap, and this difference was more efficient than the mean of intra- and interspecific sequence variability[25, 26].

In this study, the average intraspecific K2P distance was 0.21%, compared with 6.50% for species within genera. The mean interspecific distance was found to be 31-fold higher than the mean intraspecific distance, which was similar to the 25-fold difference observed in Australian marine fishes [2] and the 26.2-fold difference observed in Canadian mesopelagic and upper bathypelagic marine fishes[27]; this result corresponds to the DNA barcoding principle that interspecific divergence sufficiently outscores intraspecific divergence. In addition, the difference was greater than the 13.9-fold difference observed in the marine fishes commonly encountered in the Canadian Atlantic [28]. The amount of variation in mitochondrial DNA observed in this study can lead to demographic changes in fish populations. The mean K2P distance increased gently within the higher taxonomic ranks of families and species classes, with values of 23.70% and 25.60%, respectively. The rate of increase declines in the higher taxonomic categories due to substitutional saturation.

The entire NJ tree derived from the study is shown in Fig 2. Most species were clustered into monophyletic units in the NJ tree, indicating that DNA barcoding has high efficiency in species identification. Morphological misidentification can change the outcome of the NJ tree. We detected deep divergence of 5.99% among individuals of *Muraenesox cinereus* (MG220575, KX215195, KX215196) at first. Three sequences obtained from *M*. *cinereus* formed two different clusters. The sequence MG220575 clustered away from the rest and clustered with *Uroconger lepturus* with a K2P distance of zero. Without this single sequence, the conspecific divergence of *M*. *cinereus* was zero. We checked the preserved specimens and discovered that the single specimen was a larva of *U*. *lepturus*, which was originally classified as *M*. *cinereus*. Morphological misidentifications of voucher specimens, DNA contamination and incomplete knowledge of the taxonomic literature can contribute to ambiguous barcoding results [29, 30]. On the other hand, the reference library of barcodes and species identification requires a large number of specimens, including eggs, larvae and adults, and many morphometric characteristics change during distinct developmental stages. Therefore, occasional instances of misidentification are inevitable, and this example reflects that DNA barcoding can detect cases of morphological misidentification. The combination of morphological and molecular characteristics is a necessary condition for establishing a molecular database. A successful reference barcode library can be used to better characterize and broadly identify species.

Two other cases can be found in the NJ tree: sequences that have the same species name but do not exhibit cohesive clustering by conspecies in which detect deep divergence can be detected among them, and sequences that have different species names and form a cohesive cluster. The sequences of *Thryssa kammalensis* clustered separately in the NJ tree; one sequence showed a genetic divergence of 3.30% with others and 4.30% with *Thryssa dussumieri*, but it clustered with *Thryssa hamiltonii* with a K2P distance of zero. We rechecked the identification history of the preserved samples and found some intermediate morphological characteristics in them. The main factors responsible for this case may be introgressive hybridization. Mitochondrial genes are maternally inherited, and the hybrid would have only maternal species DNA. When species with a close phylogenetic relationship mate, the subsequent generation can have the morphological characteristics of either parent species. Introgressive hybridization would lead to phylogenetic paraphyly. The species of *Takifugu*, which formed a cohesive cluster, exhibited fairly low interspecies divergence with a value of zero and could not be discriminated by DNA barcoding. This failure was due to recent and rapid speciation, and the specimens of these species were genetically similar at the DNA barcode region. The factors of erroneous taxonomy, low sister species divergence, introgressive hybridization and the scarcity of specimens were also theoretically associated with the failure of DNA barcoding. A more rapidly evolving DNA fragment, such as the mitochondrial control region, should be used in such specimens, and nuclear genes should also be sequenced to establish whether hybridization has occurred.

Fish diversity in the Taiwan Strait is highly threatened by overexploitation, and some scientists predict that all fisheries will have collapsed by 2048[9, 31]. With the significant decline in biodiversity, species extinction enhances the need for the conservation of marine biodiversity [32]. Our results reveal that DNA barcoding was successful in identifying the vast majority of fish species. Identification supported by DNA barcoding could be used to evaluate fish biodiversity, monitor fish conservation and manage fisheries [14, 33]. This technique will provide direction for future studies of fish species that need to be barcoded. Once a fish DNA barcode database has been established, the scientific and practical benefits of fish barcoding are diverse. DNA barcoding can discriminate all fish species and identify the eggs, larvae and carcass fragments of these species. The results will provide more information on fish diversity to the fisheries managers and ecologists who craft the policies for the conservation and sustainable use of fishing resources.
